# Conceptual Overview of Biological Age Estimation

**DOI:** 10.14336/AD.2022.1107

**Published:** 2023-06-01

**Authors:** Ahmed Salih, Thomas Nichols, Liliana Szabo, Steffen E Petersen, Zahra Raisi-Estabragh

**Affiliations:** ^1^William Harvey Research Institute, NIHR Barts Biomedical Research Centre, Queen Mary University of London, Charterhouse Square, London, EC1M 6BQ, UK.; ^2^Barts Heart Centre, St Bartholomew’s Hospital, Barts Health NHS Trust, West Smithfield, London, EC1A 7BE, UK.; ^3^Wellcome Centre for Integrative Neuroimaging, FMRIB, Nuffield Department of Clinical Neurosciences, University of Oxford, Oxford, UK.; ^4^Big Data Institute, Li Ka Shing Centre for Health Information and Discovery, Nuffield Department of Population Health, University of Oxford, Oxford, UK.; ^5^Health Data Research UK, London, UK.; ^6^Alan Turing Institute, London, UK.

**Keywords:** aging, senescence, biological age, machine learning, mathematical models, brain

## Abstract

Chronological age is an imperfect measure of the aging process, which is affected by a wide range of genetic and environmental exposures. Biological age estimates may be derived using mathematical modelling with biomarkers set as predictors and chronological age as the output. The difference between biological and chronological age is denoted the “age gap” and considered a complementary indicator of aging. The utility of the “age gap” metric is assessed through examination of its associations with exposures of interest and the demonstration of additional information provided by this metric over chronological age alone. This paper reviews the key concepts of biological age estimation, the age gap metric, and approaches to assessment of model performance in this context. We further discuss specific challenges for the field, in particular the limited generalisability of effect sizes across studies owing to dependency of the age gap metric on pre-processing and model building methods. The discussion will be centred on brain age estimation, but the concepts are transferable to all biological age estimation.

## 1. Introduction

Global population aging has increased the burden from non-communicable diseases of older age. Promotion of healthy aging is a public health priority. Increased age is associated with loss of function across organ systems and increased propensity to disease [1]. However, there is variation in age-related loss of function amongst individuals of the same age. Thus, chronological age is not always a perfect measure of biological age, which is influenced by a wide array of genetic and environmental exposures throughout the lifecourse [2].

Over the last 50 years many researchers have attempted to estimate biological age as an alternate measure of the aging process. In general, the premise of these works is the development of a mathematical model for estimation of biological age using biomarkers as model predictors and chronological age as the model output. The discrepancy between the model estimated biological age and the observed chronological age is denoted as an “age gap” metric, which gives more information about the aging process over and above chronological age alone [3]. Given that aging processes may be differential across organ systems, many researchers advocate organ-specific biological age estimation.

The emergence of biomedical research databases, such as the UK Biobank [4], with availability of highly detailed phenotypic characterisation of very large samples has provided unique opportunities to develop biological age estimation models. In particular, the vast expanse of neuroimaging data has permitted development of biological brain age estimation models using image-derived phenotypes as predictor biomarkers [5]. Existing work has demonstrated the utility of brain age gap derived from these models through demonstration of associations with key environmental and genetic exposures [6]. Whilst biological age estimation is an old endeavour, it has found new applications in the field of brain age estimation [3]. There is growing interest in application of these concepts to understanding aging process of other organ systems [7].

The development and interpretation of biological age estimation models differs somewhat from that of conventional models. In this paper we review the concept of the age gap, assessment of model performance in the context of age estimation, sources of variation in biological age estimates, and limitations and future work in the field. The discussion will be centred on brain age estimation, but the concepts are broadly transferable to any biological organ age estimation.

## 2. Brain age gap

Brain age estimates provide a prediction of biological brain age based on neuroimaging phenotypes. A variety of methods can be used to estimate age as a continuous variable, including multiple linear regression [8], principal component analysis [9-11], the Hochschild’s method [12], the Klemera and Doubal’s method [13, 14], support vector regression [15], Xgboost regression [16] and Bayesian ridge regression [17]. Non-regression methods may also be used for estimation of brain age, such as deep learning and decision trees. In the context of biological brain age estimation, chronological age plays the role of the dependent variable, and brain phenotypes are the independent variables. These variables are fitted in a regression model as in the following equation 1 (for a single observation).

$$y_{i}=b_{0}+x_{i}b+\varepsilon_{i}$$ [1]

where *y_i_* is the dependent variable, *b*_0_ is the intercept, *x_i_* is a vector (i.e., brain imaging phenotypes), *b* is the coefficient value and *ε_i_* is the error. Once brain age ˆ*y_i_* has been estimated, the brain age gap is calculated by subtracting chronological age from predicted brain age, ˆ*y_i_* - *y_i_*, this is the equivalent of the residual or the error from the model (though regression residuals are usually computed instead as *y_i_* - *y*ˆ*_i_*).

The next step is a bias correction. Linear regression methods that minimise the mean squared error produce residuals that are uncorrelated with the fitted values (predicted age), but the residuals are correlated with the response variable (chronological age). Since this results in systematic over-prediction of brain age for younger subjects and under-prediction of older subjects, a bias correction is applied. While such bias corrections slightly increase the mean squared error, they remove this systematic structure from the brain age gap (see [18] for details).

The “model predicted brain age” is the conditional population mean, i.e., the mean age prediction given the covariates x. A positive brain age gap may be interpreted as a brain that is “older” and is indicative of increased risk of cognitive impairment and brain diseases. Conversely, a negative brain age gap can be considered as representing healthy or delayed brain aging [19]. The associations of brain age gap with exposures of interest may be examined to evaluate variation explained by biological brain age that is not already explained by chronological age. Thus, such association studies should include chronological age as a covariate. It is important to note, that while the bias correction method aims to ensure the age gap metric is uncorrelated with chronological age, the two metrics may be exactly uncorrelated (e.g., if analysis is performed on a held-out sample or different subset) or have non-zero partial correlation given that they will be conditional on other covariates in the model [18].

**Figure 1.**
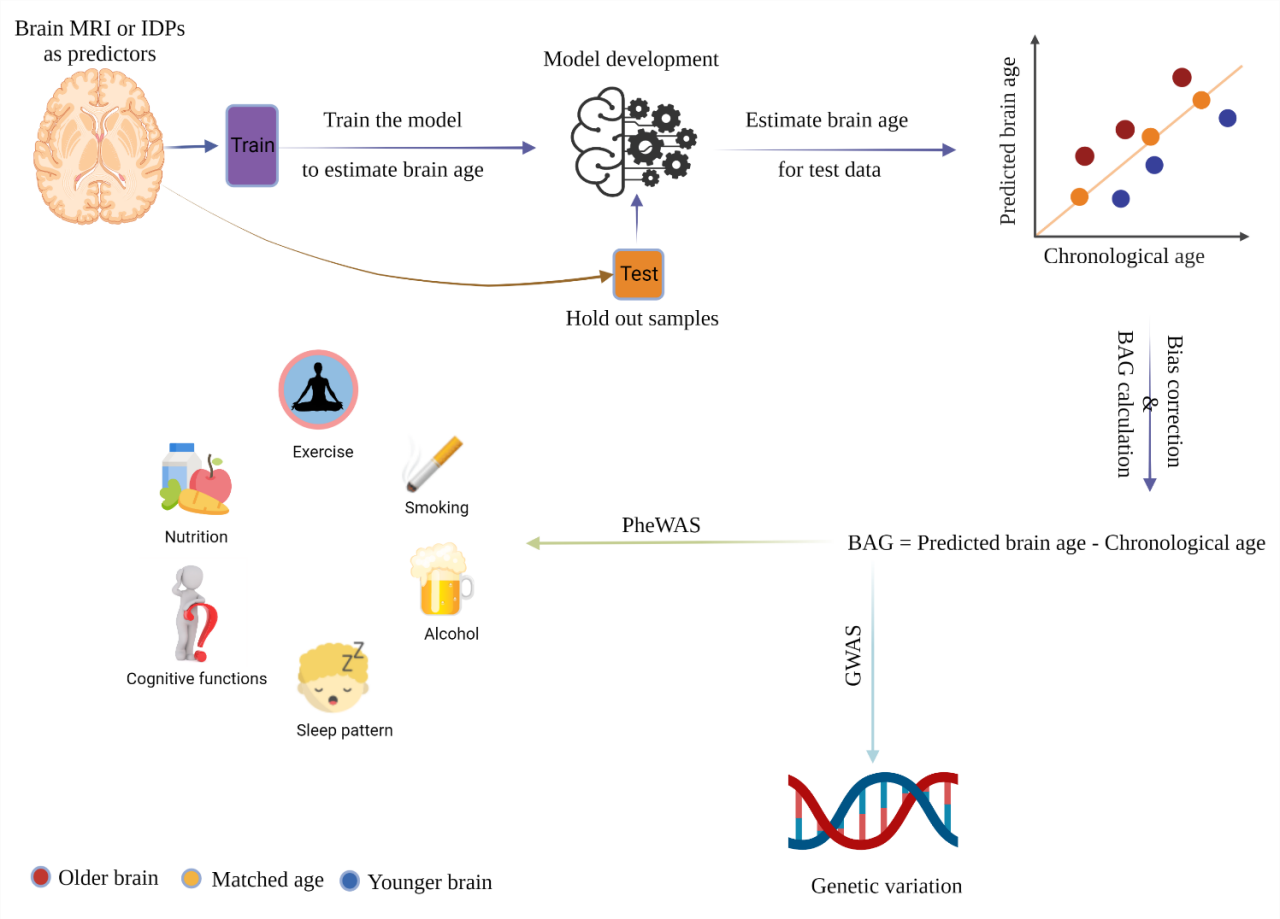
**General illustration of the brain age estimation pipeline and GWAS and PheWAS with the derived BAG.** The path starts by using either the raw brain MRI or IDPs as model predictors (input variables). The model output is chronological age. The model is developed on a training set and tested on a held-out sample. There is then a bias correction step followed by BAG calculation. BAG is calculated as the model predicted age - chronological age. The BAG can then be used as phenotype of interest (indicator of aging) in PheWAS and GWAS to examine the association of wide range of lifestyle exposures and genetic on BAG. MRI: Magnetic resonance imaging; IDPs: image derived phenotypes; BAG: brain age gap; GWAS: Genome-wide association study; PheWAS: Phenome-wide association study.

The brain age gap extracted and interpreted in this way has been used as a phenotype of interest in phenom-wide association studies (PheWAS) and genome wide association studies (GWAS) to investigate associations of demographic, environmental, and genetic exposures on brain health represented by the brain age gap [20]. These studies have contributed to establishing the biological validity of brain age gap through demonstration of associations with cognitive tests, and genetic and environmental exposures. Furthermore, examination of associations with brain age gap can provide insight into novel determinants of brain aging. Figure 1 illustrates the general pipeline for brain age estimation and examination of age gap associations.

Thus, biological age estimated in this way can be used to assess aging for any organ (brain, heart, liver, etc.) and provides an easily understandable output which can be compared against chronological age and used to investigate the associations of a wide range of exposures with advanced aging. However, the biological age estimates provided vary by the input data used, modelling methods, and sample size, which limits comparability across studies. Furthermore, in cases where complex modelling methods are used, it can be difficult to interpret the contribution of model input variables to the estimated biological age. Finally, it is challenging to evaluate model performance in the context of biological age estimation using conventional methods.

## 3. Optimal age estimation

Assessment of model performance in the context of biological age estimation requires special considerations. Evaluation of model performance quantifies the accuracy with which the model estimates the value of interest. Mean Absolute Error (MAE) is one of the most commonly used measures of model performance. MAE represents the absolute difference between the predicted value and the observed value averaged over the *n* subjects in the sample [21]. Hence, MAE quantifies the overall error in the model; it can be calculated as in equation 2

$$\text{MAE}=\frac{1}{n}\sum_{i=1}^{n}\left|y_{i}-{\hat{y}}_{i}\right|$$ [2]

where *y_i_* is the actual value, ˆ*y_i_* is the predicted value and *n* is the number of subjects to estimate their dependent variables.

In conventional model building, we regard smaller MAE values as indicative of better model performance, in that the model predicted values are close to the observed values. In the context of biological age estimation there is, by construct, no ground truth and the model with the smallest error is not necessarily the one producing the optimal age gap estimate (*ϵ*) [5].

Fundamentally, it is important to note that conventional approaches to evaluation of model performance are not always useful for assessment of biological age estimation models. The purpose of biological age estimation is to construct a measure of the aging process that would provide incremental information over that captured by chronological age. Perfect age prediction would in fact produce no useful “age gap”, in what is known as the “biomarker paradox” [14]. As such, the most useful assessment of model performance lies not in examination of metrics such as the MAE but in the strength and consistency of associations between the extracted age gap metric and key exposures of interest.

## 4. Variation in biological age estimates

Many studies have estimated biological brain age and performed PheWAS and GWAS analyses with brain age gap to uncover the effects of genetics variations and environmental exposures on brain aging [22, 20]. In these studies, brain age gap was significantly associated with many exposures that advance brain aging such as diabetes, alcohol, and smoking in a biologically consistent direction. In addition, different sets of SNPs in genes with known functional importance in brain diseases have been found to associate with brain age gap [6, 20]. The results are clear indications for validity of the method and potential of clinical translation.

Although multiple studies highlight biologically consistent associations with their brain age gap metrics, there is significant variation in the magnitude of estimates reported. For instance, the effect of diabetes on brain aging has been reported by several studies with each reporting different effect sizes. Salih, et. al [22] reported that diabetes advances brain aging by 6 months while James et al. [19] reported that diabetes advances brain age gap by 2 years. Similar differences in effect size estimates were observed for associations with smoking and alcohol consumption. Baecker et al. report increase of 3.4 years and 4.1 years in brain age gap with smoking and greater alcohol consumption, respectively [3]; while Cole et al. report smaller magnitude of associations in their analysis of brain age gap with the same exposures [19].

These differing effect sizes highlight the highly specific interpretation required for brain age gap measures produced by each analysis. There are multiple sources of variation in biological age estimation at all levels of model development and in evaluation of age gap associations. These include characteristics of the population on which the model was built, the predictor IDPs and factors related to their acquisition and post-processing, and mathematical modelling methods deployed. Furthermore, there are likely significant variations in ascertainment and definition of exposure variables which further impact the observed associations with the age gap metric. These observations highlight the importance of comprehensive and transparent reporting of all steps in development and assessment of age estimation models. Ultimately standardisation of approaches to biological age estimation will be required to permit cross comparability of relationships.

## 5. Limitations and future work

A key limitation of biological estimation methods is the lack availability of adequate datasets for model development. This is more important in cases where study of a specific cohort is required e.g., young children [23] or individuals with an uncommon illness [24]. In these settings obtaining adequate training data can be extremely challenging. Firstly, a reliably verified disease sample which adequately encompasses the full spectrum of the condition of interest is required. Second, the expected atypicality of the disease cohort means that a simple age-matched comparator cohort is likely to be insufficient with need for attention to other sample characteristics. More generally, large extensively phenotyped datasets are available almost exclusively in the context of dedicated biomedical databases such as the UK Biobank [4]. Such datasets provide a valuable platform for development of biological age estimation models as well as provide opportunity to examine patterns and determinants of aging with potential for novel insights into aging pathways. Such analyses may advance our understanding of the biology of aging and highlight exposures that may be tackled at a population level to promote healthy aging. A growing body of literature addresses these questions with regards biological brain aging. More recently, similar approaches have been taken to estimation of biological age across other organs, such as the heart [7]. Further work is required to develop optimal biological age estimation models that may be utilised to better understand the aging process within and across organ systems. A general limitation of such work is that broad generalisability of these models to other settings may be limited as extensive phenotyping in research datasets is unlikely to be widely available. A question that has not been adequately addressed is the potential clinical utility of biological age estimation, which hinges on the predicated age estimates providing information that is incremental to chronological age in terms of disease discrimination and event prediction. Increased availability of large datasets with detailed clinical phenotyping and longitudinal outcome tracking will permit evaluation of these key questions.

## 6. Conclusion

Chronological age does not always fully capture the biological age of an individual. Biological age estimation is a method of deriving an alternate measure of the aging process with the aim of providing information that is complementary to chronological age. In brain age estimation neuroimaging biomarkers are used as model predictors to predict chronological age as outcome. The discrepancy between model predicted age and chronological age is calculated to derive a brain age gap. The influence of genetic and environmental exposures on brain aging may be studied through examination of their associations with brain age gap. Optimal model performance in the context of biological age estimation is best assessed through consideration of the consistency and strength of exposure associations with the derived age gap metric. There is substantial variation in brain age estimation models due to differences in samples, predictors, and modelling methods. As such, effect sizes may not be comparable across different studies. These observations underscore the importance of exact reporting of methods and phenotypes in biological age estimation work. The concepts of brain age estimation may be applied to derive biological age estimation for other organs. Further work is required to explore the applications of this method for examining mechanistic pathways of the aging process within and across organ systems. Existing work demonstrates potential for clinical and research utility of biological age estimation. Further work is needed to establish the additional value of biological age estimates over chronological age for prediction of clinical events.

## References

[1] Ruthsatz M, Candeias V (2020). Non-communicable disease prevention, nutrition and aging. Acta Bio Medica Atenei Parm, 91(2):379 10.23750/abm.v91i2.9721 PMC7569619 32420978

[2] Jia L, Zhang W, Chen X (2017). Common methods of biological age estimation. Clin Interv Aging, 12:759. 28546743 10.2147/CIA.S134921 PMC5436771

[3] Baecker L, Garcia-Dias R, Vieira S, Scarpazza C, Mechelli A (2021). Machine learning for brain age prediction: Introduction to methods and clinical applications. EBioMedicine, 72. 10.1016/j.ebiom.2021.103600 PMC8498228 34614461

[4] Sudlow C, Gallacher J, Allen N, Beral V, … PB-Pl (2015). UK biobank: an open access resource for identifying the causes of a wide range of complex diseases of middle and old age. PLoS medicine, 12. 10.1371/journal.pmed.1001779 PMC4380465 25826379

[5] Smith SM, Vidaurre D, Alfaro-Almagro F, Nichols TE, Miller KL (2019). Estimation of brain age delta from brain imaging. Neuroimage, 200:528-39. 31201988 10.1016/j.neuroimage.2019.06.017 PMC6711452

[6] Ning K, Zhao L, Matloff W, Sun F, Toga AW (2020). Association of relative brain age with tobacco smoking, alcohol consumption, and genetic variants. Sci Reports, 10(1):1-10. 10.1038/s41598-019-56089-4 PMC6992742 32001736

[7] Raisi-Estabragh Z, Salih A, Gkontra P, Atehortúa A, Radeva P, Boscolo Galazzo I, et al (2022). Estimation of biological heart age using cardiovascular magnetic resonance radiomics. Sci Reports, 12(1):1-12. 10.1038/s41598-022-16639-9 PMC9329281 35896705

[8] Dubina TL, Dyundikova Evz VA (1983). Biological age and its estimation. II. Assessment of biological age of albino rats by multiple regression analysis. Experimental gerontology, 8:5-18. 10.1016/0531-5565(83)90046-3 6873212

[9] Bai X, Han L, Liu Q, Shan H, Lin H, Sun X, et al (2010). Evaluation of biological aging process - A population-based study of healthy People in China. Gerontology, 56(2):129-40. 19940465 10.1159/000262449

[10] Jee H, Jeon BH, Kim YH, Kim HK, Choe J, Park J, et al (2012). Development and application of biological age prediction models with physical fitness and physiological components in Korean adults. Gerontology, 58(4):344-53. 22433233 10.1159/000335738

[11] ZhangWG, BaiXJ, SunXF, CaiGY, BaiXY, ZhuSY, et al (2014). Construction of an integral formula of biological age for a healthy Chinese population using principle component analysis. J Nutr Heal Aging, 18(2):137-42.10.1007/s12603-013-0345-824522464

[12] Hochschild R (1989). Improving the precision of biological age determinations. Part 1: A new approach to calculating biological age. Exp Gerontol, 24(4):289-300. 2684676 10.1016/0531-5565(89)90002-8

[13] Cho IH, Park KS, Lim CJ (2010). An empirical comparative study on biological age estimation algorithms with an application of Work Ability Index (WAI). Mech Ageing Dev, 131(2):69-78. 20005245 10.1016/j.mad.2009.12.001

[14] Klemera P, Doubal S (2006). A new approach to the concept and computation of biological age. Mech Ageing Dev, 127(3):240-8. 16318865 10.1016/j.mad.2005.10.004

[15] Cortes C, Vapnik V (1995). Support-Vector Networks. Mach Learn, 20(20):273-97.

[16] Chen T, Guestrin C (2016). Xgboost: A scalable tree boosting system. In: Proceedings of the 22nd acm sigkdd international conference on knowledge discovery and data mining, 785-94.

[17] MacKay DJ (1992). Bayesian interpolation. Neural Comput, 4(3):425-47.

[18] Butler ER, Chen A, Ramadan R, Le TT, Ruparel K, Moore TM, et al (2021). Pitfalls in brain age analyses. Hum Brain Mapp, 42(13):4092-101. 34190372 10.1002/hbm.25533 PMC8357007

[19] Cole JH (2020). Multimodality neuroimaging brain-age in UK biobank: relationship to biomedical, lifestyle, and cognitive factors. Neurobiol Aging, 92:34-42. 32380363 10.1016/j.neurobiolaging.2020.03.014 PMC7280786

[20] Smith SM, Elliott LT, Alfaro-Almagro F, McCarthy P, Nichols TE, Douaud G, et al (2020). Brain aging comprises many modes of structural and functional change with distinct genetic and biophysical associations. Elife, 9. 10.7554/eLife.52677 PMC7162660 32134384

[21] Wang W, Lu Y (2018). Analysis of the Mean Absolute Error (MAE) and the Root Mean Square Error (RMSE) in Assessing Rounding Model. IOP Conf Ser Mater Sci Eng, 324(1).

[22] Salih A, Boscolo Galazzo I, Raisi-Estabragh Z, Rauseo E, Gkontra P, Petersen SE, et al (2021). Brain age estimation at tract group level and its association with daily life measures, cardiac risk factors and genetic variants. Sci Reports, 11(1):1-14. 10.1038/s41598-021-99153-8 PMC8523533 34663856

[23] Copeland A, Silver E, Korja R, Lehtola SJ, Merisaari H, Saukko E, et al (2021). Infant and Child MRI: A Review of Scanning Procedures. Front Neurosci, 1-16. 10.3389/fnins.2021.666020 PMC8311184 34321992

[24] Matalon Reuben and Delgado Lisvania and Michals-Matalon K (2018). Canavan disease. In: Adam MP, Everman DB, Mirzaa GM, et al., editors. GeneReviews® [Internet]. Seattle (WA): University of Washington, Seattle; 1993-2022.

